# Does muscular activity related to vertical facial divergence influence the time needed for orthodontic extrusion of palatally impacted maxillary canines? A retrospective study

**DOI:** 10.4317/jced.55149

**Published:** 2018-09-01

**Authors:** Michele Tepedino, Maciej Iancu-Potrubacz, Cristina Grippaudo, Claudio Chimenti, Giuseppina Laganà

**Affiliations:** 1 Department of Biotechnological and Applied Clinical Sciences, University of L’Aquila, L’Aquila, Italy; 2 Fondazione Policlinico Universitario A. Gemelli IRCCS, Roma-Università Cattolica del Sacro Cuore. Istituto di Clinica Odontoiatrica e Chirurgia Maxillo-facciale, Rome, Italy; 3 Department of Clinical Sciences and Translational Medicine, University of Rome Tor Vergata, Rome, Italy

## Abstract

**Background:**

The aim of the present study was to evaluate if the different muscular activity correlated to different degrees of facial divergence has an effect on the time needed to extrude a palatally impacted maxillary canine.

**Material and Methods:**

Twenty-six patients were retrospectively selected, all treated with a specific cantilever appliance that allows extrusion of the impacted canine applying a physiologic amount of force below 0.6 N in a predictable way. For all the patients, pre-treatment cephalometric tracings were used to evaluate facial divergence through the FMA angle, the angle between the maxillary and mandibular plane, and the angles between the occlusal plane and either the maxillary and mandibular plane. Linear bivariate regression was calculated to evaluate if facial divergence can predict the time needed for canine extrusion.

**Results:**

The linear regression model was not able to predict extrusion time from variables explaining the facial divergence.

**Conclusions:**

Palatally impacted maxillary canines can be treated with the application of physiologic extrusion force regardless of patients’ facial divergence and muscular activity.

** Key words:**Impacted canines, cantilever, facial divergence, muscular activity.

## Introduction

The impaction of maxillary canines can occur in nearly 2% of the population (1), more specifically 2.4% in the italian population (2), being caused by either genetic or environmental factors (3-5). When an alteration in the eruption pathway is early intercepted, an interceptive treatment comprising extraction of the deciduous canine(1) and rapid maxillary expansion (6), possibly anchored on deciduous teeth (7,8), can be performed. Nevertheless, in many cases orthodontists have to mechanically erupt an impacted canine. In most of the cases, the impacted canine is palatally displaced (85% of the cases), requiring a treatment that is usually complex and time-consuming (9). In fact, the treatment of a malocclusion that comprises an impacted tooth requires more time than a similar malocclusion without impaction (10), and needs a complex planification because the selection of the surgical technique, the modality of orthodontic traction, the arch space management, and the preparation of the anchorage should be carefully planned (11-13). All the enlisted factors should be addressed to achieve a satisfactory functional and aesthetical result, avoiding complications such as root resorption and loss of vitality of impacted or neighboring teeth (14-16).

To improve the efficiency of orthodontic treatment of palatally impacted maxillary canines, several techniques and appliances have been proposed, including Kilroy springs, double archwires, powerchains, magnets, and cantilevers (17). Most of these systems, as highlighted in an article by Yadav *et al.* (18), produce high forces, around 2.5 N, that exceed the recommended threshold of 0.6 N(14), resulting in a higher risk of complications. In two previous articles, we proposed a cantilever system that allows the use of light physiologic force in a predictable way, with the advantage of having a device made out of stainless steel (19,20).

While it is known that orthodontic treatment is generally followed by a muscular and functional adaptation (21-23), the effects of masticatory muscles associated with different kinds of tooth movements should be evaluated during treatment planning (24). For example, strong bite forces are observed in subjects with parallel jaws and augmented posterior face height, while weaker bite forces are associated with long-face patients (25), both in adults and children (26), and these aspects influences orthodontic tooth movements (27). In addition, since the device used for extrusion of maxillary impacted canines is located in the palatal vault, the effect of the tongue should be also considered: the tongue has the ability to deliver orthodontic forces and to move teeth (28), and different tongue and hyoid bone positions have been observed in hypodivergent and hyperdivergent subjects (29) that can result in different magnitude of tongue pressure (30).

The purpose of this study was to evaluate the effect of the different muscular and tongue patterns in hypodivergent and hyperdivergent patients on the time needed to extrude a palatally impacted canine with a device that predictably produces an amount of force around 0.6 N. The null hypothesis was that facial divergence has no effect on the time needed to extrude the impacted tooth.

## Material and Methods

The present research protocol was approved by the Internal Review Board of the University of L’Aquila (Protocol number 23169). The records of patients treated for the orthodontic extrusion of an impacted maxillary canines at the Dental clinic of the Department of Biotechnological and Applied Clinical Sciences, University of L’Aquila from January 2007 to January 2018 were retrospectively screened for the following inclusion criteria:

- Unilateral or bilateral canine impaction with a palatal displacement;

- Orthodontic traction performed with a calibrated amount of force through a previously described cantilever appliance (19);

- No failure of the traction (debonding of the canine’s bracket, breakage of the ligature, etc.); reported in patient’s clinical history that could have affected extrusion time;

- Absence of local or systemic conditions that could alter bone metabolism and tooth movement.

Sample size calculation (G*Power version 3.1.9.2, Universitat Dusseldorf, Germany) (31) revealed that for a linear bivariate regression with an α error of 0.05, a power of 0.8, and a calculated slope of 0.36, a sample of 26 subjects was needed. Therefore, the first 26 subjects in chronological order that met the inclusion criteria were included in the study sample.

**Figure 1:**
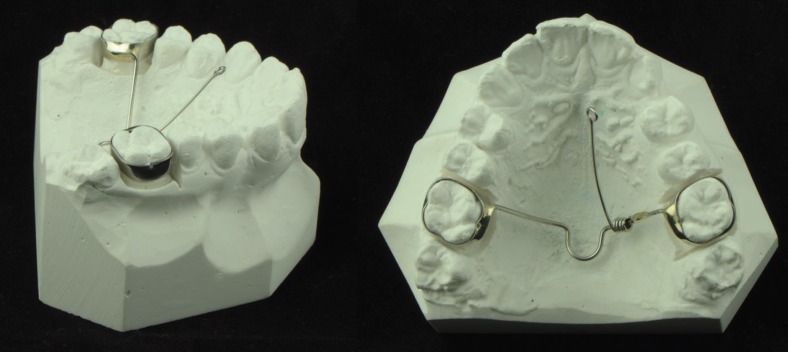
The cantilever appliance used to orthodontically extrude the palatally impacted canines in the present study.

**Figure 2:**
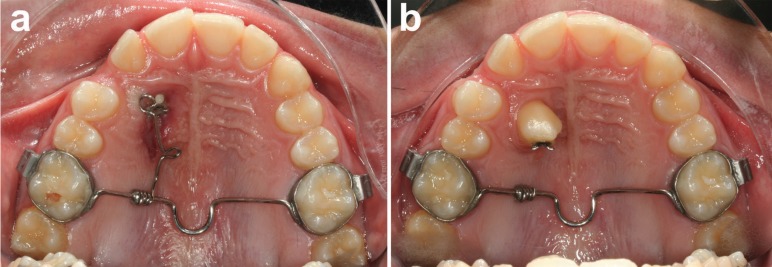
An example of the cantilever appliance used to extrude an impacted canine.

All patients had palatally impacted canines extruded with a specific device described in a previous work (19): the appliance was made of a 0.9 mm stainless steel transpalatal arch with a distal loop welded to two molar bands for the upper first molars, and a 0.6 mm stainless steel cantilever welded to the transpatal arch and then rolled around it to create 5 loops. The cantilever was pre-activated to have its end at approximately 15 mm from the point of force application on the crown of the impacted canine (Figs. 1,2). The singularity of the described appliance is that with such a configuration it is possible to predictably deliver to the impacted canine a physiologic force not exceeding 0.6 N.

**Figure 3:**
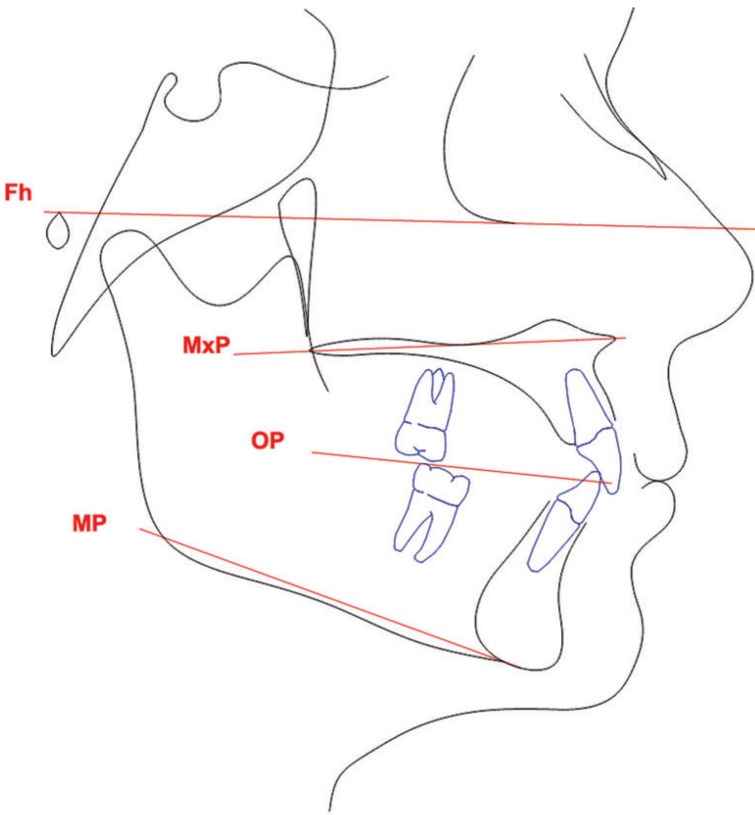
Reference planes used to evaluate the facial divergence. Fh, Frankfurt plane; MxP, maxillary plane; OP, occlusal plane; MP, mandibular plane.

For all the selected patients, orthodontic extrusion time (from the moment when the cantilever was first tied to the impacted tooth until the moment when the cantilever was removed because the canine had reached the occlusal plane) was retrieved from the patient’s record; in the case of bilateral impaction, the extrusion time of the two canines was averaged to have a single variable. In addition, pre-treatment lateral cephalograms were collected. The following cephalometric variables (Fig. 3) were calculated for every patient:

- FMA, the angle between the Frankfurt plane and the mandibular plane;

- MP-MxP, the angle between the mandibular plane and the maxillary plane passing through the anterior and posterior nasal spine;

- MP-OP, the angle between the mandibular plane and the occlusal plane;

- MxP-OP, the angle between the maxillary plane and the occlusal plane.

Tracings were performed by an expert operator (MT) and repeated after a two-weeks interval. An Intra-Class Correlation (ICC) coefficient was calculated between the two set of measurements to evaluate the intra-operator reliability.

-Statistical Analysis

Descriptive statistics were calculated for all the variables. A linear bivariate regression was calculated to predict orthodontic extrusion time from amount of facial divergence, as explained by the selected cephalometric angles. Normal P-P Plots were also used to check the assumption of homoscedasticity and normality of residuals. First-type error was set as 0.05. Statistical analysis was carried out using SPSS software (SPSS for Windows, Version 13.0. Chicago, SPSS Inc.).

## Results

Regarding the error of the method, the calculated ICC coefficient was excellent (> 0.85) for all the variables, revealing good intra-observer reliability of the measurements.

**Figure 4:**
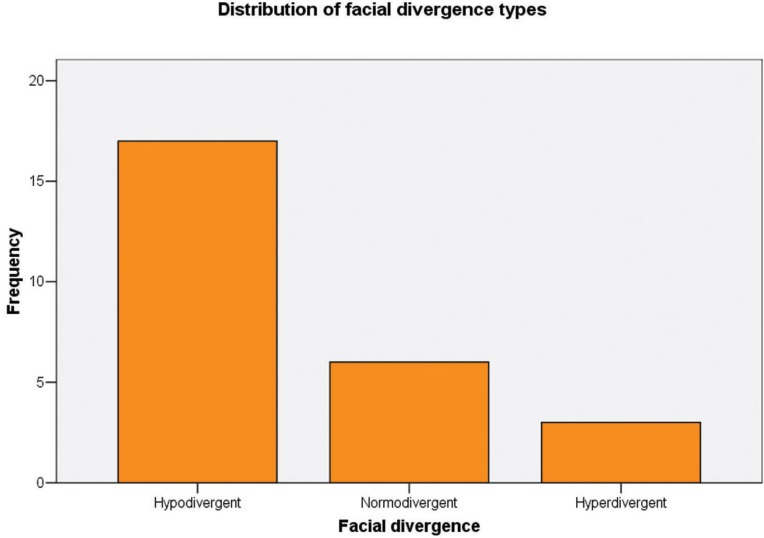
Frequencies of patients classified as normodivergent, hypodivergent, or hyperdivergent according to their FMA value.

Descriptive statistics are reported in  Table 1. According to the FMA values, 17 patients could be classified as hypo-divergent (65.4% of the total sample), 6 patients as normo-divergent (23.1%), and 3 (11.5%) as hyper-divergent (Fig. 4).

**Table 1:**
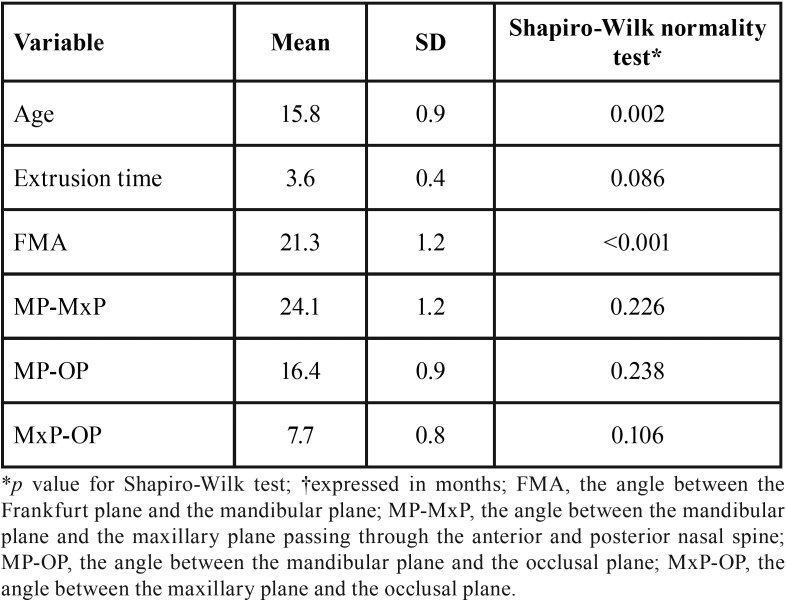
Descriptive statistics for patients’ age, orthodontic extrusion time and cephalometric variables (n= 26).

The correlation matrix for the studied variables is reported in  Table 2. The variable MP-MxP was excluded from subsequent analysis because of a strong (>0.7) correlation with FMA and MP-OP, to avoid multicollinearity of the data.

**Table 2:**
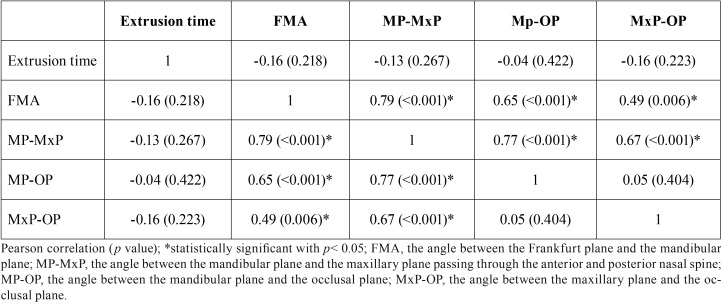
Correlations between orthodontic extrusion time and cephalometric variables explaining facial divergence (n= 26).

The linear regression model was not able to explain a relation between extrusion time and facial divergence ( Table 3), therefore the null hypothesis was accepted.

**Table 3:**
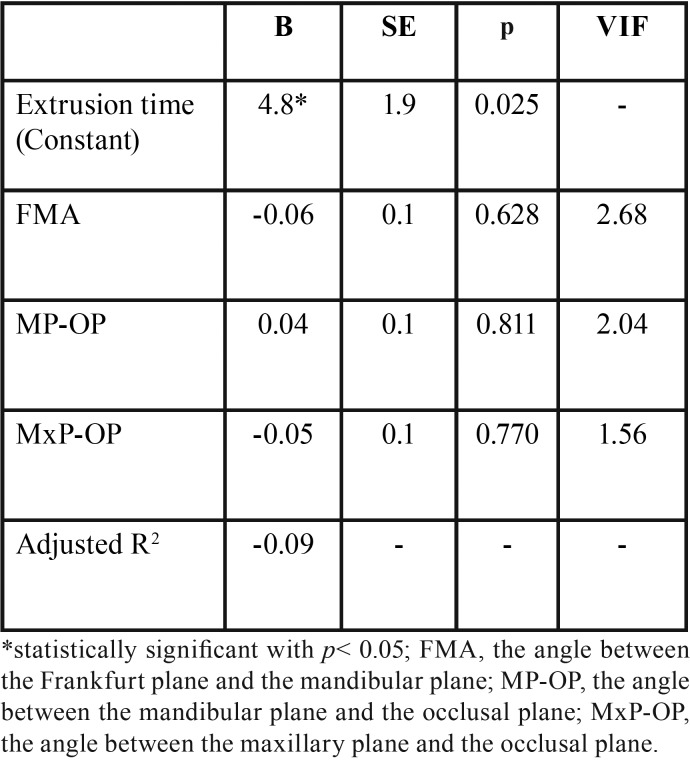
Linear bivariate regression outcome (n= 26).

## Conclusions

Applying a physiologic force for the orthodontic extrusion of palatally impacted canines with a specially designed cantilever appliance resulted in a mean traction time of 3.6 months. This treatment time was not explained by cephalometric variables describing the patient’s divergence; therefore, palatally impacted canines can be successfully treated with the described protocol regardless of the patient’s vertical skeletal and muscular pattern.
